# Molecular Characterization and Comparative Genomics of Clinical Hybrid Shiga Toxin-Producing and Enterotoxigenic *Escherichia coli* (STEC/ETEC) Strains in Sweden

**DOI:** 10.1038/s41598-019-42122-z

**Published:** 2019-04-04

**Authors:** Xiangning Bai, Ji Zhang, Anoop Ambikan, Cecilia Jernberg, Ralf Ehricht, Flemming Scheutz, Yanwen Xiong, Andreas Matussek

**Affiliations:** 1 Division of Clinical Microbiology, Department of Laboratory Medicine, Karolinska Institutet, Karolinska University Hospital, Huddinge, Sweden; 2 State Key Laboratory of Infectious Disease Prevention and Control, National Institute for Communicable Disease Control and Prevention, Chinese Center for Disease Control and Prevention, Beijing, China; 3 mEpiLab, New Zealand Food Safety Science & Research Centre, Institute of Veterinary, Animal and Biomedical Sciences, Massey University, Massey, New Zealand; 4 The Public Health Agency of Sweden, Solna, Sweden; 5 InfectoGnostics Research Campus e.V., Philosophenweg 7, Jena, Germany; 6 Leibniz Institute of Photonic Technology e.V. Jena (Leibniz-IPHT), Jena, Germany; 7 The International Centre for Reference and Research on Escherichia and Klebsiella, Unit of Foodborne Bacteria and Typing, Department of Bacteria, Parasites and Fungi, Statens Serum Institut, Copenhagen, Denmark; 8 Karolinska University Laboratory, Stockholm, Sweden; 9 Department of Laboratory Medicine, Region Jönköping County, Jönköping, Sweden

## Abstract

Hybrid *E. coli* pathotypes are representing emerging public health threats with enhanced virulence from different pathotypes. Hybrids of Shiga toxin-producing and enterotoxigenic *E. coli* (STEC/ETEC) have been reported to be associated with diarrheal disease and hemolytic uremic syndrome (HUS) in humans. Here, we identified and characterized four clinical STEC/ETEC hybrids from diarrheal patients with or without fever or abdominal pain and healthy contact in Sweden. Rare *stx2* subtypes were present in STEC/ETEC hybrids. Stx2 production was detectable in *stx2a* and *stx2e* containing strains. Different copies of ETEC virulence marker, *sta* gene, were found in two hybrids. Three *sta* subtypes, namely, *sta1*, *sta4* and *sta5* were designated, with *sta4* being predominant. The hybrids represented diverse and rare serotypes (O15:H16, O187:H28, O100:H30, and O136:H12). Genome-wide phylogeny revealed that these hybrids exhibited close relatedness with certain ETEC, STEC/ETEC hybrid and commensal *E. coli* strains, implying the potential acquisition of Stx-phages or/and ETEC virulence genes in the emergence of STEC/ETEC hybrids. Given the emergence and public health significance of hybrid pathotypes, a broader range of virulence markers should be considered in the *E. coli* pathotypes diagnostics, and targeted follow up of cases is suggested to better understand the hybrid infection.

## Introduction

*Escherichia coli* strains isolated from intestinal diseases have been grouped into at least six main pathotypes on the basis of epidemiological evidence, phenotypic traits, clinical features, and specific virulence factors^1^. The well-described intestinal pathotypes of diarrheagenic *E. coli* (DEC) are Shiga toxin-producing *E. coli* (STEC), enterotoxigenic *E. coli* (ETEC), enteropathogenic *E. coli* (EPEC), enteroaggregative *E. coli* (EAEC), enteroinvasive *E. coli* (EIEC), and diffusely adherent *E. coli* (DAEC). The virulence-associated genes that are unique to a pathotype have been used as molecular markers to define the pathotype of *E. coli* strains.

STEC, defined by the production of phage-encoded Shiga toxins (Stxs), poses a significant public health concern as it can cause a wide spectrum of symptoms ranging from asymptomatic carriage to severe diarrhea, as well as bloody diarrhea and hemolytic uremic syndrome (HUS)^1^. Stxs are classified into two major families, Stx1 and Stx2 (encoded by *stx1* and *stx2*) on the basis of toxin neutralization assays and sequence analysis^2^. The Stx1/Stx2 have been further classified into three Stx1 subtypes (Stx1a, Stx1c, and Stx1d) and seven Stx2 subtypes (Stx2a to 2 g) in *E. coli* strains based on sequences similarities and phylogenetic analysis^2^. Stx subtypes display dramatic differences in disease causing potency^3^. Stx2a (with or without Stx2c) and Stx2d are regarded to be more potent than other subtypes and highly associated with HUS^4^. However, the clinical significance of other Stx subtypes as well as the interplay of other multiple virulence-associated factors has been noted^5,6^. Notably, a novel Stx2 subtype, named Stx2h was identified from wild marmots recently, which exhibited a hybrid virulence genes spectrum and pathogenic potential^7^. ETEC, which is defined by the presence of the plasmid-encoded heat-labile (LT) and/or heat-stable toxins (ST)^8^, has been identified as a major cause of significant diarrheal illness worldwide^9,10^. The two classes of ST, STa (encoded by *sta*) and STb (encoded by *stb*), differ in sequences and mechanism of action^8^. STa is associated with human disease, while STb is typically associated with infection in pigs, although *stb* has been found in human ETEC isolates^8^.

Many virulence markers for a pathotype are often carried on mobile genetic elements (MGEs) such as phage, plasmid as mentioned in STEC and ETEC, thus allowing acquisition of virulence genes via MGEs and horizontal gene transfer leading to the emergence of hybrid pathotypes. Certain Stx-phages can infect and lysogenize almost all known *E. coli* pathotypes, including both DEC and extraintestinal pathogenic *E. coli* (ExPEC)^11^. The hybrid STEC pathogens are causing infections and outbreaks in many countries, with the most notorious hybrid being the STEC/EAEC strain O104:H4, which caused a large outbreak with numerous HUS cases and deaths in Germany in 2011^12^. Other STEC hybrid types, for instance, STEC/ExPEC O80:H2 hybrid has been reported to cause HUS and bacteremia^13^, STEC/UPEC hybrids have been identified from hospitalized patients^14,15^. Hybrid types like EPEC/EAEC and ETEC/EPEC have also been reported from patients^16,17^. STEC/ETEC hybrids have been recovered from various sources including humans, animals, food, and water^18–21^, and some STEC/ETEC strains have been associated with diarrheal disease and HUS in humans^22,23^.

In Sweden, the prevalence of STEC in hospitalized patients with diarrhea is around 1.2%-1.8% according to previous studies^24,25^. But little data is available concerning the presence of emerging hybrid pathotypes and its correlation with illness. Here, we present the identification and characterization of STEC/ETEC hybrid strains from Swedish diarrheal patients with or without fever or abdominal pain and healthy contact. The molecular properties of these strains were investigated by initial microarray analysis followed by whole genome sequencing (WGS). The phylogenomic analysis was used to assess the phylogenetic position of these hybrids among a diverse collection of *E. coli* and *Shigella spp*. representing all major pathotypes. Based on these results, we discuss the potential public health importance of these hybrid *E. coli* strains.

## Results

### STEC/ETEC hybrids in Diarrheal Patients and Clinical Features

Four out of 195 clinical STEC isolates (2.05%) over a 15 years-period investigation in Region Jönköping County, Sweden, were found to carry both *stx2* and *sta*, designated as STEC/ETEC hybrid pathotype. The four hybrids were isolated from individuals infected in local cities in Sweden where they were living. Three hybrids were from diarrheal patients, among which one had abdominal pain and fever simultaneously. One strain was from an individual sampled by contact tracing, and without any clinical symptoms included in this study. The duration of *stx* shedding was available in three patients, which ranged from 11 to 65 days. The four STEC/ETEC hybrids and associated clinical features are listed in Table 1.

**Table 1:** Characteristics of STEC/ETEC hybrid strains in this study.

Strain	Serotype	*stx* subtype	*sta* subtype	ST	Sampling year	Clinical symptom	Duration of *stx* shedding (day)	Age of patients (year)
SE572	O187:H28	*stx2g*	*sta4*, *sta5*	200	2005	D	11	1
SE573	O15:H16	*stx2g*	*sta4*	325	2009	D, AP, F	16	56
SE574	O136:H12	*stx2a*	*sta4*, *sta4*, *sta5*	329	2014	N	18	10
SE575	O100:H30	*stx2e*	*sta1*	993	2017	D	—	82

D: Diarrhea. AP: Abdominal pain. F: Fever. N: No symptoms, individual was sampled due to contact tracing around an index case. -: Unavailable.

### Genome Assemblies of STEC/ETEC Hybrids

All four STEC/ETEC genomes remained as draft genomes comprising of 244, 393, 552, 350 contigs, respectively. The chromosome sizes, coding DNA sequences (CDSs), rRNA and tRNA found in these hybrids are summarized in Table 2.

**Table 2:** Genome features of STEC/ETEC hybrid strains in this study.

	SE572	SE573	SE574	SE575
Coverage	46	79	82	31
No. contigs	244	393	552	350
Total length (bp)	5 266 892	5 380 960	5 520 089	5 012 207
G + C ratio (%)	51%	50%	50%	51%
No. CDS	5 088	5 149	5 297	4 755
No. rRNA	8	11	10	9
No. tRNA	83	95	98	72
No. Prophages	11	12	12	9
Accession number	SAMN09758572	SAMN09758573	SAMN09758574	SAMN09758575

### Serotypes, *stx* Subtypes, Virulence Genes and Sequence Types

Four serotypes, i.e., O187:H28, O15:H16, O136:H12 and O100:H30 were assigned. The four hybrids carried *stx2* genes, which were assigned with three *stx2* subtypes: two strains from diarrheal patients carried *stx2g*, one from a diarrheal patient harbored *stx2e*. Strain SE574 without any investigated-symptoms which was sampled from contact tracing possessed *stx2a* (Table 1**)**. Except *stx2* and *sta* genes, other virulence factors were identified in these hybrids which mainly belonged to three categories: toxin, adherence factor and serum resistance (Table 3). The four hybrids belong to different MLST sequence types (ST200, ST325, ST329 and ST993) (Table 1**)**. Serotypes, *stx* subtypes, virulence genes determined by whole genome sequences analysis matched the initial microarray analysis.

**Table 3:** Presence of virulence genes carried on four STEC/ETEC hybrids.

Category	Gene	Product/Function	SE572	SE573	SE574	SE575
Toxin	*stx2*	Shiga toxin 2	+	+	+	+
	*sta*	Heat-stable enterotoxin STa	+	+	+	+
	*astA*	EAEC heat-stable enterotoxin I	+	+	+	−
	*cba*	Colicin B	−	+	+	−
	*cma*	Colicin M	−	+	+	−
	*ehxA*	EHEC hemolysin	+	−	+	−
Adherence	*lpfA*	Long polar fimbriae	+	+	+	−
Serum resistance	*iss*	Increased serum survival	−	+	+	+

### Heat-stable Enterotoxin Phylogeny

**Figure 1:**
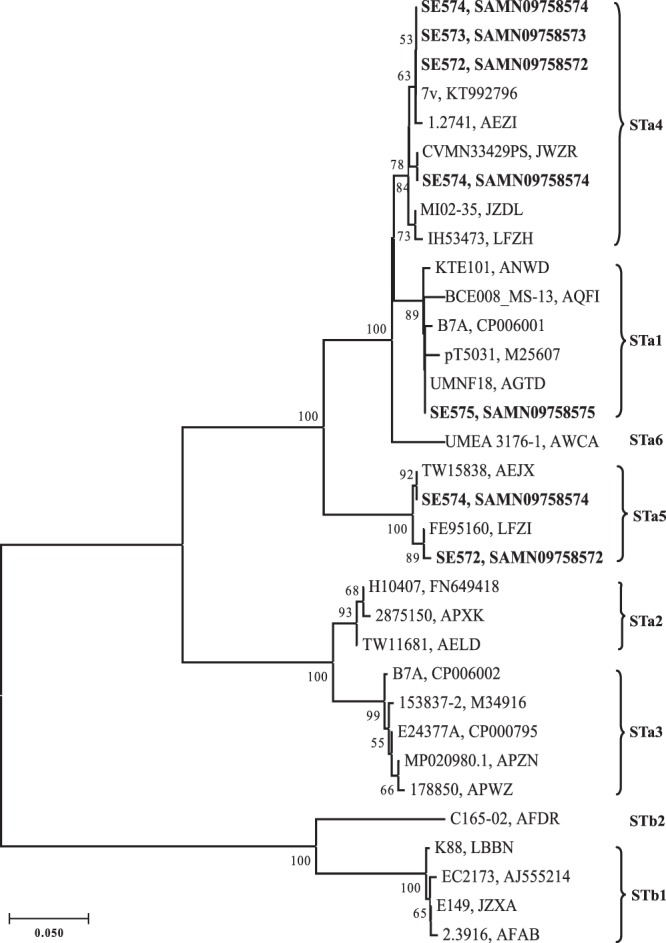
Phylogenetic tree of *sta* alleles by the neighbour-joining method. The neighbour-joining tree was inferred from nucleotide sequences of all *sta* alleles using a *p* distance matrix. Strain designations and GenBank accession numbers (or WGS prefixes) are given at the branch tips. Bootstrap values based on 1000 replications (>50%) are given at the internal nodes. *sta* subtypes are given next to the outer brackets. The four STEC/ETEC hybrid strains in this study were highlighted in bold.

Strain SE574 carried three distinct copies of *sta* gene, among which two copies exhibited high nucleotide identity (99.1%), while shared only 88.2% identity with the remaining one. SE572 carried two divergent copies of *sta* with 87.3% nucleotide identity. SE573 and SE575 possessed one copy of *sta* each. A phylogenetic scheme was further used to assign *sta* subtypes. The phylogenetic relatedness of 21 reference *sta* gene sequences assigned in this study were consistent with that reported previously^26^ (Fig. 1). The seven copies of *sta* gene carried on four hybrids were assigned as three different *sta* subtypes (*sta1*, *sta4* and *sta5*). Strain SE572 and SE574 carried both *sta4* and *sta5*, with two *sta4* copies present in SE574. SE573 and SE575 possessed *sta1* and *sta4*, respectively (Fig. 1).

### Prophage Regions, Stx-converting Phages and Stx production

For strain SE572, 11 prophage regions were identified, of which 7 regions were intact, 2 were incomplete, and 2 were questionable. Stx2g was identified in an intact phage region, where Stx phage-specific genes encoding the integrase, transcriptional regulator, antirepressor, antitermination protein Q and lysis were present. For strain SE573, 12 prophage regions were identified, of which 7 regions were intact, 4 were incomplete and 1 was questionable. Nevertheless, Stx2-converting phage on SE573 remained unidentified as no Shiga toxin gene was found by using PHAST tool. For strain SE574, 12 prophage regions were identified, of which 6 were intact, and 6 were incomplete. Stx2a was found on an incomplete phage region, where Stx phage-specific genes encoding antirepressor and antitermination protein Q were present. For SE575, 9 prophage regions were identified, of which 5 were intact, and 4 were incomplete. Stx2e was found on an incomplete phage region, where antitermination protein Q was observed. The Stx-phages insertion sites of these hybrids remained unidentified due to the limitation of draft genomes used. Stx2 production was detectable in one *stx2a*-containing strain, while undetectable in two *stx2g*-containing strains by both the Duopath® STEC Rapid Test and Vero cell assay. For the *stx2e*-containing strain, Stx was detectable by VCA while not by Duopath® STEC Rapid Test.

### Plasmid-associated Sequences

PlasmidFinder indicated several plasmid replicon sequences of known Inc groups in four STEC/ETEC genomes (Table 4). Strain SE572, SE573 and SE574 had three, four and five plasmid replicons, respectively. SE575 had two plasmid replicons: IncFII (pHN7A8) and IncI1 (Alpha). Notably, the plasmid-associated gene *sta* was placed in the same contig as IncFII in strain SE575. In addition, according to genome annotation of SE575, several plasmid-associated genes, such as plasmid segregation protein ParM, plasmid stability protein, IncFII RepA protein family protein, replication regulatory protein RepB were located in the same contig as *sta* gene. In the plasmid assembly sequences generated with plasmidSPAdes algorithm, one copy of *sta* gene was identified in each of the plasmid sequences of strain SE572, SE573 and SE574.

**Table 4:** Plasmid replicons found in the four STEC/ETEC hybrids.

Strain	Replicon	Reference Acc.	Coverage (%)	Identity (%)
SE572	IncFIB	AP001918	100	98
	IncFII	AY458016	100	99
	Col156	NC_009781	100	94
SE573	IncFIB	AP001918	100	97
	IncI2 (Delta)	AP002527	100	98
	IncFII (pCoo)	CR942285	100	96
	Col (MG828)	NC_008486	100	92
SE574	IncFIB	AP001918	100	97
	IncFII (pSE11)	AP009242	100	92
	IncI1 (Alpha)	AP005147	100	100
	IncB/O/K/Z	GU256641	98	95
	Col156	NC_009781	100	94
SE575	IncFII (pHN7A8)	JN232517	100	98
	IncI1 (Alpha)	AP005147	100	98

### Phylogenetic Position of STEC/ETEC Hybrids

**Figure 2:**
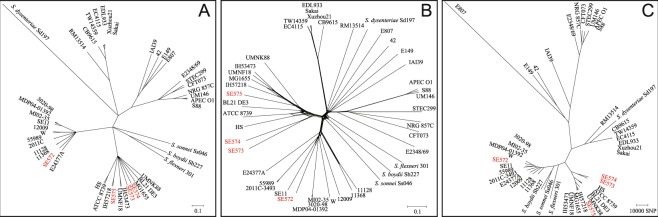
Phylogenetic relationships of STEC/ETEC hybrid strains in this study to other *E. coli*/*Shigella* reference strains. The phylogenetic positions of the four hybrid strains in relative to the other 41 reference strains were studied with three different approaches: panel (A) 80% consensus tree generated from three runs of 55 ribosomal protein subunits (*rps*) gene ClonalFrame analysis; panel (B), Neighbor-Net phylogeny inferred from the allele profiles of the 2181 loci that shared by the 45 isolates; and panel (C) Gubbins tree of the concatenated sequences of the shared loci that found in the wgMLST analysis. The four hybrid isolates were highlighted with red letter.

A ClonalFrame tree (Fig. 2A) was inferred from 55 concatenated ribosomal protein gene sequences that are single-copy and shared by 45 strains, which revealed that the four hybrids in this study formed two clusters. Three hybrid strains SE573, SE574 and SE575 were grouped together with previously characterized STEC/ETEC hybrids IH53473 and IH57218 from human, UMNF18 from pig, ETEC strain UMNK88, laboratory-adapted and commensal *E. coli* strains. Strain SE572 clustered together with strains comprising of ETEC (E24377A), STEC (11128, 11368), EAEC (55989), STEC/ETEC hybrids (3020-98, MDP04-01392 and MI02-35), STEC/EAEC hybrid (2011C-3493), laboratory-adapted and commensal *E. coli*. Similarly, neighbour-net phylogeny (Fig. 2B) and Gubbins tree (Fig. 2C) generated with concatenated sequences of 2181 shared loci found in wgMLST analysis were also consistent with this finding. Our analysis indicted that despite different *E. coli* pathotypes are inter-mixed, for example, STEC and ETEC genomes can be found nearly all branches, STEC/ETEC hybrid strains showed more tendency to cluster together.

## Discussion

STEC/ETEC hybrids have been identified from animals and humans in Finland^22^. Here, we report four STEC/ETEC hybrid strains among 195 clinical STEC strains in Sweden over a 15 years-period investigation. The incidence of STEC/ETEC hybrids among clinical STEC collection was 2.05%. In this study, microarray analysis was initially used for molecular characterization of all STEC strains, the results in terms of serotypes, *stx* genotypes and virulence genes were consistent with the in-depth WGS analysis for these hybrids subsequently. It has been suggested that microarrays, while still faster and cheaper than WGS, might serve as a pre-screening tool to define candidates worth sequencing for better understanding of molecular features associated with pathogenicity and the prospective detection of emerging pathogens^27^. Currently, hybrid DEC pathotypes remain unrecognized in some local clinical microbiological laboratories where WGS is not routinely performed, and/or where other pathogenic DEC markers and combinations are not looked upon in analysis pipelines due to the surveillance strategies and diagnostics capacities. Our study suggested that more genetic determinates should be included in the STEC or other DEC pathotypes diagnostic, and hybrids pathotypes should be take into account the surveillance and patient care. Furthermore, information obtained in this study contributes significantly to a better understanding of epidemiological and genomics characteristics of hybrid *E. coli* strains.

Stx2a subtype was reported to be highly associated with severe disease outcomes such as HUS^1^. In our study, the *stx2a*-positive strain SE574 was isolated from an individual around an index case, and without any clinical symptoms investigated, indicating the role of other virulence or host-related factors in STEC pathogenesis. Stx2e is closely associated with oedema disease in pigs, and only sporadic in humans with uncomplicated diarrhea or asymptomatic carriers^28,29^. Nevertheless, Stx2e-producing STEC strains have been isolated from patients with acute diarrhea and HUS^30,31^, thus the clinical significance of rare human *stx* subtype shouldn’t be neglected. One hybrid strain carrying *stx2e* and *sta* was isolated from an 82-year-old diarrheal patient, to the best of our knowledge, this is the first report of clinically relevant STEC/ETEC hybrid that harbors *stx2e* subtype. Stx2g was originally identified in bovine *E. coli* isolates^32^, a low prevalence of *stx2g* was reported in humans^33^. In our study, two out of four STEC/ETEC hybrids carried *stx2g*, one was from a 56-year-old diarrheal patient who had fever and abdominal pain simultaneously. Our findings accorded with a German study^33^, where all of the *stx2g-*carrying isolates from patients with diarrhea, fever and abdominal pain, were classified as STEC/ETEC hybrid. Notably, our *stx2g-*carrying strain SE573 from a diarrheal patient with fever and abdominal pain exhibited the same serotype O15:H16 and same sequence type ST325 as the German strains^33^. STEC/ETEC strains harboring *stx2a* and *stx2e* subtypes produced Stx2, while no detectable Stx2 production was observed in the two *stx2g-*containing strains in this study. Our data accords with a previous report^33^ that Stx2g was not expressed in some STEC/ETEC hybrid strains. In additional, Garcia-Aljaro *et al*.^34^, Beutin *et al*.^35^, and Miko *et al*.^36^ also reported *stx2g*-positive strains lacking any Stx expression. Notably, the Stx detection by the two methods used in this study implied the potential false-negative result by the commercial product which might fail to detect some Stx subtypes.

ETEC virulence marker STa (*sta*), is associated with human disease. Six *sta* subtypes (*sta1*-*sta6*) have been designated, among which *sta4* was more frequently found especially in patients with diarrhea and HUS^26^. In our study, different *sta* subtypes/combinations were found, three out of four hybrids harbored *sta4* subtype, with one carrying two non-identical copies of *sta4*, indicating that *sta4* subtype might be more clinically relevant. Notably, we found one strain carried three different copies of *sta* gene, one possessed two distinct *sta* copies. STa are found predominantly on plasmids^37^. In our study, the *sta* genes were identified in the plasmid assemblies of strain SE572, SE573 and SE574. In strain SE575, plasmid-related genes, such as incFII family plasmid replication initiator gene *repA*, surrounded the *sta* gene. These findings suggested that the *sta* genes in the four hybrid strains were strongly associated with plasmid and were likely horizontally transferred through plasmid, which was in consistent with the findings in the phylogenetic analysis.

In addition to *stx* and *sta*, the four hybrids harbored other virulence genes, some of which have been associated with ETEC, STEC, and EAEC strains. For instance, three strains were positive for *astA* encoding EAEC heat-stable enterotoxin I, which can be present among strains of STEC, EAEC, EPEC, ETEC, and EIEC pathotypes^38^ and even ExPEC^14^. Nevertheless, except the long polar fimbriae gene *lpfA*, which was present in three strains, none of well-defined adherence-associated genes, such as *eae*, *efa1*, *iha* was identified in our hybrid strains. Moreover, the four hybrids were all negative for ETEC colonization factors, as was the case with STEC/ETEC hybrids reported in Finland^39^. It is not uncommon that ETEC strains are negative for ETEC colonization factors^40^, thus other or some novel colonization-associated factors remain to be further explored.

Both the virulence factors and serotypes could be utilized in risk assessment and epidemiological surveillance. The four hybrids belonged to rare serotypes, interestingly, two out of four O groups O15 and O136, but with different H types, have previously been described as STEC/ETEC hybrid strains^33^. It remains to be further investigated, when more hybrid strains are collected, if some serogroups/serotypes or clones share similar genomic backbone that are more likely to acquire virulence genes, leading to emergence of hybrid pathotypes.

The phylogenetic placement of our clinical STEC/ETEC hybrids demonstrated close relatedness with certain ETEC, STEC/ETEC hybrids, STEC, EAEC, laboratory-adapted and commensal *E. coli* strains, which was in agreement with previous finding^39^. It is not surprising that STEC/ETEC hybrids, STEC or ETEC strains scattered in different phylogenetic groups. The dramatic plasticity of the *E. coli* genome accelerates the adaptation of this species into various environments, which provides numerous opportunities for new variants to emerge via the gains and losses of genes^41^. The Stx-phages and their ability to transfer genes horizontally play an important role in the evolution of *E. coli* and development of hybrid pathotypes^42^. In addition, the plasmids carrying ST/LT toxin genes *sta*/*stb* can be transferred between *E. coli* strains^43^. Commensal *E. coli* strains may also evolve to be pathogenic strains as certain parts of their genomes may act as genetic repositories for virulence factors^44^. Our analysis implied that horizontal transmission of *stx2a* and/or *sta* genes by the independent acquisition of the phages and/or plasmids carrying these genes may lead to the emergence of STEC-ETEC hybrids.

It has been indicated that the *E. coli* genome seems to be formed by an “ancestral” and a “derived” background, each one responsible for the acquisition and expression of different virulence factors^45^. In this study, we found evidence that STEC/ETEC hybrid strains may have similarities in their genetic background. By combining our data with the previously sequenced STEC/ETEC genomes, we observed that our clinical STEC/ETEC strains clustered with human STEC/ETEC strains O101:H33 IH53473 and O2:H27 IH57218 isolated from HUS and diarrheal patients, respectively in Finland, and both of strains possesses *stx2a* and *sta4*^26^. Besides, our strains were phylogenetically close to some animal-derived STEC/ETEC hybrids, such as strain O147:H4 UMNF18, which was recovered from a pig with diarrhea and possessed *sta1* and *stx2e*^26^. It has been noted that certain genetic background is required for the acquisition and/or maintenance of virulence genes located on MGEs^45^, and further effort is needed to unveil the structure and characteristic of particular genetic background that are more easily to pick up other genes leading to hybrid pathotypes. No host-specific cluster was observed among hybrid strains in this study, which might partly be due to the limited number of STEC/ETEC genomes available from different sources. Strain SE572 isolated from an infant with diarrhea, formed a separate cluster together with ETEC O139:H28 E24377A, STEC outbreak strain O26:H11 11368 and O103:H2 12009, indicating the genetic diversity of these hybrid strains.

In conclusion, this is the first report of clinical STEC/ETEC hybrids in Sweden. The hybrids exhibit a considerable diversity in terms of virulence genes/genotypes, serotypes and genetic background. Rare *stx* subtypes and serotypes are found in these hybrids which might be associated with diarrheal disease. To the best of our knowledge, this is also the first report of clinically relevant STEC/ETEC hybrid that carry the *stx2e* subtype. Given the emergence of hybrid pathotypes may lead to serious consequences to public health, a wider range of virulence markers should be included in the *E. coli* pathotypes diagnostics. Furthermore, hybrid pathogens should be considered in the epidemiological surveillance and patient care.

## Materials and Methods

### Ethical considerations

All STEC strains and clinical data of STEC patients are collected through routine praxis used for the STEC diagnostics and surveillance performed in Region Jönköping County, Sweden. Formal consent is not required, and no specific ethical permit is needed to characterize the strains.

### Clinical Data Collection

Clinical data of STEC infected patients were collected through a questionnaire and by reviewing medical records as part of the routine infection control measures in Region Jönköping County as described previously^24^. Clinical manifestations included were diarrhea, bloody diarrhea, abdominal pain, vomiting, fever and HUS. STEC-positive patients were sampled weekly until they were negative, and the duration of *stx* shedding was defined as the time from the first positive sample to the first negative sample.

### Isolates Characterization and Determination of STEC/ETEC Hybrid Status

All STEC isolates recovered from stools of diarrheal patients in Region Jönköping County, Sweden from 2003 to 2017 were initially subjected to three different microarrays with the *E. coli* SeroGenoTyping AS-1 Kit, ShigaToxType AS-2 Kit and *E. coli* PanType AS-2 Kit, respectively, to determine serotypes, *stx* allele/subtypes and virulence genes as previously described^46^. Bacterial DNA was extracted from overnight culture with the EZ1 DNA Tissue Kit on an EZ1 instrument (Qiagen, Germany), according to the manufacturer’s instructions. Initial serotyping was also performed with O (O1 to O187) and H (H1-H56) antisera by agglutination in micro titer plates using antisera (SSI Diagnostica, Denmark). STEC strains that harbored ST encoding genes *sta*/*stb* were designated as STEC/ETEC hybrid pathotype. Production of Shiga toxin was determined by the Duopath® STEC Rapid Test according to the manufacturer’s instructions (https://www.sigmaaldrich.com/catalog/product/mm/104156?lang=en&region=SE), as well as the Vero cell assay at Statens Serum Institut (Copenhagen, Denmark).

### Whole Genome Sequencing and Assembly

Bacterial genomic DNA was extracted as described in microarray analysis. Sequencing was performed according to Sequencing by Synthesis (SBS) technology on the Illumina HiSeq2500 platform at SciLifeLab (Stockholm, Sweden) with 100 bp paired-end reads. The sequencing reads were quality-control processed and quality evaluated with QCtool pipeline (https://github.com/mtruglio/QCtool). The processed reads were assembled *de novo* with SPAdes (version: 3.12.0) in ‘careful mode’^47^. The assemblies were annotated with Prokka (version 1.11)^48^.

### Determination of Serotypes, *stx* subtypes, Virulence genes and MLST Sequence Types

The assemblies were compared to the VirulenceFinder database (DTU, Denmark) (http://www.genomicepidemiology.org/) using BLAST + v2.2.30^49^ to determine *stx* genotypes and presence of virulence genes. The cut-off values for gene identity and alignment coverage were set to 90% in the VirulenceFinder database searching. Serotype was determined by comparing assembly sequences to the SerotypeFinder database using BLAST + v2.2.30. MLST was calculated by comparing assembly sequences to the allele reference database using BLAST according to the Warwick *E. coli* MLST scheme (https://enterobase.warwick.ac.uk/species/ecoli/allele_st_search).

### Heat-stable Enterotoxin Phylogeny

The *sta* gene sequences were extracted from the assembly sequences according to the gene function prediction annotated by Prokka and further verified by comparing with the published *sta* gene sequences. Twenty-one unique reference *sta* sequences assigned as six *sta* subtypes and five unique *stb* sequences as outgroup were kindly provided by Dr. Susan R. Leonard^26^. The full nucleotide sequences (~219 bp) of *sta* gene were aligned using the ClustalW algorithm in the MegAlign module of the Lasergene software package (DNASTAR Inc.). The resulting alignment was imported into MEGA 7.0 for neighbour-joining analysis using a *p* distance matrix and 1000 bootstrap replications.

### Identification of Prophages and Plasmid-Associated Sequences

The prophages in the STEC/ETEC genomes were identified by using PHAST tool (http://phaster.ca/)^50^. The Stx-converting phage sequences were extracted from predicted prophages and then manually verified and corrected. The gene adjacent to the phage integrase was designated as the phage insertion site^18^. To detect *in silico* possible plasmids in genomes, the assemblies were used as query to search against a database of reference plasmid replicon sequences with BLAST^49^. The reference sequence database was downloaded from the Center for Genomic Epidemiology server (https://cge.cbs.dtu.dk/services/data.php). The BLAST search in reference plasmid replicon sequences were reported according to the guidance of the PlasmidFinder database (alignment coverage to the reference sequence > = 66%, and identity percentage >= 85%)^51^. To extract plasmid sequences from the WGS data, the processed sequence reads were assembled separately with plasmidSPAdes algorithm^52^ that incorporated into the SPAdes assembler (version: 3.12.0)^47^ and then annotated with Prokka^48^.

### Phylogenetic Analysis

To generate a high-resolution phylogenomic tree depicting position of STEC/ETEC hybrids, the draft genomes were compared with eight previously reported STEC/ETEC hybrid genomes and 33 representative *E. coli*/*Shigella* genomes representing all the major *E. coli* pathotypes (Supplementary Table S1). The relationships of these isolates were assessed with three different approaches: ribosomal protein subunits (*rps*) gene sequence analysis, whole-genome multilocus typing (wgMLST) and whole-genome phylogeny analysis. The *rps* gene sequences have strong clonal signal and are rich in genetic variations thus being ideal target for bacterial phylogenetic relationship characterization^53^. The complete coding sequences (CDS) of the *rps* genes shared by the 45 isolates were extracted with fast-GeP. The completed whole-genome sequence of the strain EDL933 (Acc. CP008957.1) was used as reference genome to perform an *ad hoc* fast-GeP analysis^54^. The extracted *rps* gene sequences were analysed independently three times with ClonalFrame (version 1.1) and an 80% consensus tree was converged and merged from the outputs of the three runs^55^. *Ad hoc* wgMLST analysis was performed using EDL933 as reference genome by fast-GeP^54^. A NeighborNet phylogenetic network was calculated from the wgMLST allele profiles and displayed with Splits Tree 4^56^. Whole-genome phylogeny was inferred from concatenated CDSs of the shared loci found and aligned by the fast-GeP pipeline with Gubbins analysis^57^.

### Nucleotide Sequence Accession Numbers

The draft genome sequences of the four STEC/ETEC strains SE572, SE573, SE574 and SE575 were deposited in GenBank under the accession numbers given in Table 2.

## Supplementary information

Supplemental Table S1

## Data Availability

All data generated or analysed during this study are included in the manuscript and the Supplemental Materials.
